# Involving an individual with lived‐experience in a co‐analysis of qualitative data

**DOI:** 10.1111/hex.13188

**Published:** 2021-03-31

**Authors:** Laura Hemming, Daniel Pratt, Peer Bhatti, Jennifer Shaw, Gillian Haddock

**Affiliations:** ^1^ Division of Psychology and Mental Health School of Health Sciences University of Manchester Manchester UK; ^2^ Manchester Academic Health Sciences Centre Manchester UK; ^3^ Greater Manchester Mental Health NHS Foundation Trust Manchester UK

**Keywords:** forensic, Patient and public involvement, qualitative, thematic analysis

## Abstract

**Background:**

People with lived‐experience of the phenomenon under investigation are seldom involved in the analysis of qualitative data, and there exists little guidance for those wishing to involve contributors at this stage of research.

**Aims:**

To critically reflect on the process of involving people with lived‐experience in a thematic analysis and to offer direction to other researchers.

**Methods:**

An individual with lived‐experience of residing in prison contributed to a co‐analysis of qualitative data using thematic analysis. This paper reports on involvement at each stage of a thematic analysis and follows an established reporting checklist.

**Results:**

A number of challenges and benefits were encountered in the process of co‐analysing the data. These are discussed in relation to previous research and how to overcome the challenges encountered.

**Conclusions:**

The paper concludes by giving recommendations and guidance for future researchers wishing to involve people with lived‐experience in qualitative data analysis.

## 1 INTRODUCTION

The UK’s National Institute for Health Research advisory group, INVOLVE, defines patient and public involvement as ‘doing research “with” or “by” people who use services rather than “to”, “about” or “for” them’.^1^ Importantly, *involvement* is different from *engagement*, where information and knowledge about research is provided and disseminated and also differs from *participation* where people take part in a research study as a participant.^1^

It is generally agreed that there are three main arguments for the involvement of service users in the research process.^2^ The first is an epistemological argument which suggests that involving contributors in research may bridge the gap between the direct experiences of participants and the researchers’ interpretations.^3^, ^4^ The second argument is a moral argument which focusses on the obligation of researchers to ask those most affected by the outcomes of research what research should focus on, rather than allowing ‘elite’ groups such as scientists, funders and the government to make these decisions.^5^, ^6^ Finally, there is a consequentialist argument which suggests that involving service users in the research process can lead to a higher quality, more efficacious body of research.^2^, ^7^

INVOLVE assert that there are three main levels of involvement that can be conducted.^1^ The least interactive of these levels is ‘consultation’ where members of the public are asked to give their views, which are used to inform decision making. The next level, ‘collaboration’, denotes an ongoing partnership between academic researchers and contributors, with decisions about the research being shared. The final level, user‐controlled research, describes research which is actively controlled, directed and managed by service users and service user organizations. It is important to note that research projects may adopt a combination of these approaches in practice.

In addition to the degree of control that contributors have over research, there are also recommendations regarding at which stages contributors should be involved in research. The INVOLVE briefing notes recommend that where possible, patients and the public be involved at every stage of the research cycle.^8^ This includes the following: identification and prioritization of research; commissioning of research; design and management; undertaking; dissemination; implementation; and evaluation of impact.

Despite INVOLVE recommending the involvement of contributors across several stages of research, it has been reported that contributors tend to be involved less frequently in particular stages of research such as the analysis stages. It is not clear whether this is due to a lack of reporting or because researchers are not sufficiently engaging in patient and public involvement (PPI) during this stage of a project.^9^, ^10^, ^11^ Some have suggested that research teams may be reluctant to involve people with lived‐experience in qualitative analysis due to the time and resources required to appropriately and effectively engage contributors at the analysis stage^12^, ^13^ combined with a perception that contributors do not have much to offer to the analysis stage of research.^14^

Contrary to this, it has been reported that involving people with lived‐experience in qualitative analysis can lead to several benefits. For instance, a collaborative approach to qualitative analysis has seen contributors help to; provide an extra dimension to data analysis with alternative perspectives given on themes and trends^11^, ^15^, ^16^; identify themes that are most relevant to patients or the public and may have otherwise been missed^17^, ^18^; check the validity of conclusions and correct misinterpretations^19^, ^20^; and challenge perceptions of researchers and change the way results are described in reports.^21^

Unfortunately, there currently exists a lack of guidance on how best to involve people with lived‐experience in a collaborative data analysis. This can present a barrier, particularly to inexperienced or novice researchers such as those conducting doctoral research, in involving people with lived‐experience during the analysis phase of qualitative research. This is important as arguably early career researchers are at the vanguard of involvement work in research^22^ with some arguing that PPI should be an integral part of doctoral study.^23^

This paper aims to review how best to involve people with lived‐experience in qualitative analysis. The paper will draw on the experiences of the first author, who completed a collaborative qualitative analysis as part of her doctoral study. The paper will aim to outline benefits and challenges associated with this and provide recommendations for other researchers wishing to involve contributors in a thematic analysis.

For simplicity, this paper will refer to the doctoral student and her supervisors as ‘academic researchers’, though acknowledging that these individuals may also have disclosed or non‐disclosed relevant lived‐experience. Similarly, this paper will refer to the PPI member as a ‘contributor’, though acknowledging that this individual may also have significant research skills and experience relevant to this project.

## 2 METHODS OF PATIENT AND PUBLIC INVOLVEMENT

Section 2 of this paper outlines the methods of patient and public involvement (also see Appendix B) whilst simultaneously reporting the results of a thematic analysis.

### 2.1 Study overview

The reflections presented in this paper were drawn from a study which aimed to explore how male prisoners experience alexithymia and how this relates to experiences of suicide and violence.^24^ Alexithymia can be defined as an inability to identify or communicate emotions^25^ and has been found to relate both to suicide^26^, ^27^ and to violence.^26^, ^28^, ^29^, ^30^, ^31^

Fifteen male prisoners from two prisons in the North West of England each took part in a qualitative interview. Participants were eligible to take part in this study if they had experienced custodial suicidal and/or violent thoughts and/or behaviours and if their responses on the Toronto Alexithymia Scale^32^ indicated the likely presence of alexithymia (ie total score of 52 or above). Participants were interviewed using a flexible open‐ended topic guide and were also given the opportunity to use pen and paper to draw their emotions during the interview. Interviews were audio‐recorded and transcribed verbatim.

The data were analysed using inductive thematic analysis to identify common themes and discrepancies in the data.^33^ Thematic analysis is a flexible method for identifying, analysing and reporting patterns (themes) within data.^33^ Braun & Clarke (2006) identify six phases of thematic analysis that can be completed as a recursive process whereby researchers move back and forth between phases as required. Specifically, the phases involved are familiarization, initial codes, themes, review themes, define and name themes and report writing. Additionally, a polytextual thematic analysis was conducted with the drawings created by participants.^34^

### 2.2 Recruiting and training contributors

This study is one of several studies comprising a body of research submitted as a doctoral thesis. A group of seven contributors, all with previous experience of residing in prison, advised on all of the studies within the thesis. These contributors were recruited, via existing social networks, charity organizations and local probation teams. However, it was decided to involve only one contributor in the analysis phase of the current study. This was due both to limited resources available to the doctoral student and to encourage a greater depth of involvement, avoiding tokenistic input and allowing for a rich co‐production of analysis. The choice of contributor depended on several factors including availability of the contributor, previous experience with qualitative research and previous attendance at group meetings. The contributor was paid for the time given to the project in line with INVOLVE’s recommended rate of £15 per hour. Travel expenses were also covered for the contributor.

The contributor was a 67‐year‐old male with a BA (Hons) degree in modern middle eastern history. He had previously served a five‐year sentence in prison, during which time he became a listener—a prisoner trained by the Samaritans to help other prisoners at risk of suicide and self‐harm. Prior to this study, the contributor had previous experience of co‐producing thematic analysis on other mental health research projects. LH is a 29‐year‐old female with a BSc (Hons) degree in Psychology. She has worked in mental health research for 6 years and is currently completing a PhD in psychology and mental health. LH has had a wealth of previous of experience of leading and contributing to thematic analyses on a range of mental health‐related research projects.

The contributor attended a three‐hour bespoke training session, created and delivered by LH. This included a PowerPoint presentation which contained an overview of qualitative research methods, study‐specific research questions and a step‐by‐step guide to completing a thematic analysis. First, the principles of qualitative research were discussed, including the aims, characteristics and types of research deemed ‘qualitative’. Next, the training briefly alluded to the various types of qualitative analysis that exist, highlighting that this study would utilize a Braun and Clarke‐guided thematic analysis.^33^ The training then briefly outlined the concept of constant comparison.^35^ Next, the training gave an overview of stages of a thematic analysis, stressing that these did not need to be followed sequentially. Each of these stages was then explained in more detail, including practical examples of how contributors could be involved in each stage. Examples of coded extracts were shown as well as examples of thematic maps, to provide concrete displays of thematic analysis in action. The training session concluded with a practice example, where the contributor was asked to code a 3‐page transcript and then feedback the codes they had created.

### 2.3 Involving people with lived‐experience in a Braun and Clarke‐guided thematic analysis

#### 2.3.1 Familiarization and reflexivity

The first phase outlined in Braun and Clarke's thematic analysis is familiarizing yourself with the data.^33^ It is advocated that analysts immerse themselves in the data, until they are familiar with the depth and breadth of the content. This usually involves repeated reading of transcripts, ensuring to read the data in an ‘active way’ by searching for meanings and patterns.

After all interviews were transcribed, a single transcript was chosen for focus. The option was given for the contributor to read or listen to an audio file of the interview, in an attempt to recognize the difficulties that some may face with literacy skills. However, the contributor stated that they would prefer to read the transcript than listen to it. The contributor and LH therefore independently repeatedly viewed the transcript and associated drawings, until they felt familiar with the data.

Reflexivity in the research context refers to ‘the process of critically reflecting on the knowledge we produce, and our role in producing that knowledge’. (Braun & Clarke, 2013, p.37). Specifically, personal reflexivity is the act of bringing the researcher into the research, and making us visible as part of the research process.^36^ This is important in the context of public contributors as they are likely to bring different perspectives and experiences than those of academic researchers to the research, and it has been documented that such perspectives may lead to alternative interpretations of data.^11^, ^15^, ^16^ It is therefore important for both academic and lay researchers to acknowledge their role within knowledge production, and how this relates to their previous experiences, opinions and attitudes.

In light of this, whilst familiarizing themselves with transcripts and drawings, both LH and the contributor made reflective notes. Reflective notes were encouraged to be free and wide ranging, though a document was provided to help guide reflexivity. This included questions (Appendix A) that both LH and the contributor could ask themselves during their reading of transcripts, to ensure they were reading transcripts in an ‘active’ way. These questions were adapted from other resources^36^ and combined with additional questions posed by LH. It was stressed, however, that these questions should only be used as a guide.

Comparing LH’s and the contributor's reflexive notes, there were some interesting differences. Most notably, and as expected, the contributor more frequently recalled their own experiences which they felt resonated with the experiences of the participant. Whilst LH had not made any notes in relation to questions 7 (‘Anything that resonates with your own experiences?’) and 8 (‘Anything that is very different from your own experiences?’), the contributor had listed here a range of experiences both similar to and contrasting to the participant's experiences. This was further emphasized in responses to question 13 (‘How would you feel if you were in that situation?’), where the contributor's notes corresponded to times he had actually been in similar situations, where LH’s notes detailed a hypothetical response to being in similar situations, albeit outside of a prison setting. In contrast, LH had made more extensive notes to question 17 (‘What does the interview tell you about the interview as an interactive process?’) by drawing on pauses, laughs and tone of speech to reflect on the moods and attitudes of both the researcher and the participant.

#### 2.3.2 Initial codes

The next phase of a thematic analysis is coding the data.^33^ In this phase, sections of data are highlighted which appear interesting to the analyst, to which codes are then applied. A code refers to ‘the most basic segment, or element, of the raw data or information that can be assessed in a meaningful way regarding the phenomenon’ (Boyatzis, 1998, p.63).^37^ Analysts are encouraged to work systematically through the entire data set to identify interesting aspects in the data that may later form the basis for themes.

Once the contributor and LH were familiar with the transcript and drawings, they began coding the data. The contributor chose to assign codes to a printed transcript using a combination of coloured pens and notes in the margin to identify codes. LH separately coded the transcript and drawings, using Microsoft Word's comment function to record codes. Upon meeting to discuss the codes ascribed to the transcript, the contributor identified seventeen codes and LH identified twenty‐one codes.

As can be seen from Table 1, there was a large degree of overlap in the codes ascribed to the transcript. Thus, it was possible to merge the codes that LH and the contributor had identified so that a total of seventeen codes were applied to the transcript. Interestingly, there were only two codes (‘can't listen to others emotions’ and ‘role of system in suicide’) out of the final seventeen codes that were identified by only one analyst. Of note, both were codes that the contributor had identified, and LH had not.

**TABLE 1:** Combined codes

Combined code	The contributor's original code(s)	LH’s original code(s)
Releasing emotions	No‐cut off point for emotions ➔ hurting self / others	Releasing emotions
Loss of control	Loss of control Emotions take over (lack of thoughts) Becoming angry because upset	Emotional overload / lots of emotions at once Emotions build and build ➔ out of control ➔ dual harm
Physical displays of emotion	Easier to express emotions physically than verbally	Emotions and physical responses Non‐verbal communication of emotions
Avoid looking weak	Fears of others seeing weakness—stand your ground Banter ➔ violence because thinks being made fun of	Emotions = weakness. Related to gender.
Mask vulnerable emotions	Appears normal when suicidal Vulnerable emotions ➔ self‐isolating	Masking emotions—only show in private, for example crying Avoids talking about emotions
Show confidence	Lowered control of some emotions (eg hostage taking)	More willing to show some emotions than others, for example happy / anger vs. sad / frightened
Positive / negative spectrum	Drawings ‘happy’ = positive, others = negative	Positive—negative spectrum
Impact of prison on emotions	Suicidal thoughts only developed in prison Prison makes you institutionalized Deep remorse for offence Wants to stay in prison or die	Impact of prison on emotions
Emotions and cognitions	Emotions take over (lack of thoughts)	Emotions and memories / events Emotions and thoughts
Recognizing emotions	Self‐realization—know he's angry	Identifying emotions in self Identifying emotions in moment vs. on reflection Cognitive struggle to understand emotions—difficult to talk about them
Being frightened of own emotions	Restraint = calming, cannot explode anymore	Frightened of own emotions
Who you can trust with emotions	Old‐school officers preferred not touchy feely	Emotions and others Who you can talk to RE: emotions
Learnt behaviour	Always struggled to show / express emotions before prison Family upbringing—crying and displays of affection not okay, anger is okay Chose not to engage with support early on	Learning about emotions growing up
Can't listen to others emotions	Cannot listen to others emotions / vulnerability	
Talking about emotions ➔ feeling weird	Feels weird after discussing emotions	Talking about emotions does not help
Stored anger	Holds grudges	Emotions last a long time
Role of system in suicide	Were drugs issued by healthcare staff or inmates?	

This coding framework was then applied to a second transcript by both the contributor and LH which led to further refinement of the coding framework. LH then applied the adapted coding framework to the remaining thirteen interviews, meeting with the contributor regularly to discuss revisions. Where disagreements occurred between LH and the contributor, consensus was reached by exploring other excerpts of data identified under the same code and by refining and adapting the coding framework.

#### 2.3.3 Themes and reviewing themes

Once all data have been coded and collated, the next phase of thematic analysis is to search for themes.^33^ Here, analysts should sort codes into potential themes and collate all coded extracts within each theme. Braun & Clarke (2006) encourage the use of visual methods at this phase, such as tables, mind‐maps and organizing pieces of paper into theme piles. Analysts should also create a candidate thematic map in this phase which details the relationship between codes, themes and different levels of themes.

**FIGURE 1:**
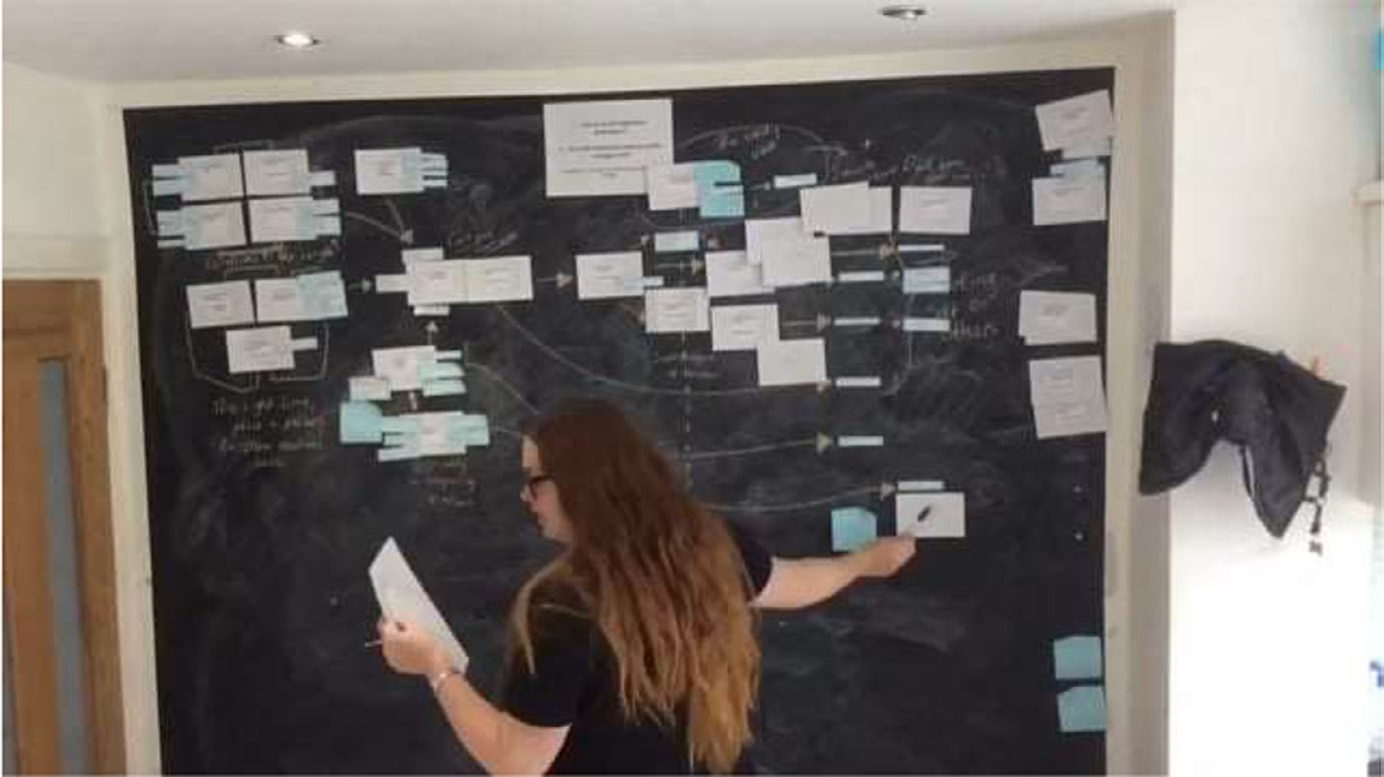
Searching for themes

The contributor and LH therefore worked through each code systematically, first excluding any codes which they felt did not directly relate to the research question. Next, they began grouping together clusters of codes which they felt related to one another. This process is illustrated in a time‐lapse video below (Figure 1; Video S1).

Where possible, the contributor led the discussion of codes. This meant that the contributor input to the decisions on creating themes was central and guided much of the analysis. Crucially, though the process of searching for and reviewing themes was led by academic researchers, the content that formed the initial and final themes was co‐produced and reflected equal input from both the contributor and academic researchers. Where there was disagreement, LH and the contributor discussed the code in great detail before coming to a decision on its place in the thematic map.

Once a candidate set of themes has been devised, the next phase is to review and refine those themes.^33^ Thus, the candidate themes were reviewed by the academic researchers and these suggestions were then agreed with the contributor.

#### 2.3.4 Define and name themes and report writing

Phases 5 and 6 of a thematic analysis involve defining and naming themes and writing the report.^33^ They are presented as one section here, due to the overlap between the two phases. Braun & Clarke (2006) note that once a thematic map has been established, it is important to be able to clearly define what each theme is and what it is not. Furthermore, at this phase working titles of themes should be replaced with concise, punchy titles which immediately give the reader a sense of what the theme is about. Finally, a report should be produced which gives a concise, coherent, logical, non‐repetitive and interesting account of the data. Braun & Clarke (2006) advise that authors should provide sufficient quotes from the data to support the themes, and where possible quotes should be vivid, straight‐forward accounts of the concept being explained by the theme.

LH produced a first draft of the methods and results sections of the report. The contributor was involved in giving extensive, specific input into this section by advising on the naming of themes and subthemes and by choosing quotes most representative of themes and subthemes. This was achieved by LH inserting several eligible quotes which could represent each theme/subtheme. The contributor was invited to rank these in terms of which they most felt should be included in the report. The contributor was also asked to rank these based on: how easy the quote was to understand; quotes which were emotive / jumped out at you more; succinctness of the quote; and how many times that participant has already been quoted.

## 3 REFLECTIONS

There was a large degree of overlap in the codes that the contributor and LH assigned to the data. Such an overlap in codes could indicate an alignment between the academic researcher and the contributor in their quest to highlight particular themes within the data set. Previous studies which have co‐analysed data with this population^38^ have also found an overlap in the codes ascribed to the data, and this may therefore be reflective of the power imbalance existing between academic and lay researchers, suggesting that co‐produced knowledge is not as achievable within such boundaries.^39^ It has been noted that it may be easy to lose contributors’ voices during multiple coding, as the process relies heavily on the willingness to listen, debate and concede.^11^ Detailed notes taken at each meeting which recorded the nature of discussions that took place appeared to suggest a balanced contribution to the discussion, with the contributor leading the discussion wherever possible. Furthermore, in an attempt to redress power imbalance, a great level of rapport was built between the contributor and LH. The building of this relationship may have been made possible by the contributors’ involvement starting early on in the research project, which others have noted can lead to more will and enthusiasm from contributors about being involved in analysis stages.^9^, ^40^ Indeed, early involvement in the research project may help to give contributors a greater sense of ownership over the project which itself can help to redress issues of power imbalance with academic researchers.

Another way of redressing the power imbalance is to involve more than one contributor. This could have had theoretical benefits, which may have led to more nuanced views of the data, with more rigorous and dynamic debate. Indeed, others have noted that focus groups, as opposed to individual interviews, can lead to a richer and more complete understanding of the topics under question.^41^, ^42^, ^43^ Unfortunately, the involvement of only one contributor in the present analysis does limit the extent to which the recommendations given here can be generalized to other studies. Future studies wishing to involve people with lived‐experience in the analysis stages should therefore budget appropriately to allow the same depth of involvement as has been detailed here, with two or more contributors.

Previously, some people have raised concerns about providing training to contributors as doing so may risk ‘professionalizing’ them, and dilute the lived‐experience brought to the research study.^44^ The training given in the present study was perceived by the academic researchers to be both necessary and beneficial, providing a sound base from which contributors could apply analytic skills. Additionally, the contributor felt that the training was adequate and covered all training needs. Further, the training given did not appear to ‘dilute’ any lived‐experience which the contributor bought to the analysis, since the contributor was frequently able to draw upon his own experiences and compare these to experiences recounted by participants. Further, the contributor noted feeling worried about their ability to complete the analysis, and the training given was thought to strengthen confidence in their analytical skills. This, again, may have gone some way to redressing the power imbalance between academic and lay researchers.

In addition to providing academic support to contributors, it is important to note the emotional impact that contributors may experience as a result of being involved in qualitative analysis. For instance, the present study invited the contributor to recount experiences of suicide, violence and incarceration as part of the analysis, all of which may have been distressing. In line with previous research which has involved people with lived‐experience,^45^, ^46^, ^47^ a number of provisions were put in place to ensure the well‐being of both academic researchers and the contributor. Namely, academic researchers who were clinically trained were always contactable at times of meetings, to ensure that any emotional distress was handled appropriately. Future researchers involving people with lived‐experience in analysis of potentially distressing datasets are encouraged to consider and mitigate the emotional impact of this on contributors.

### 3.1 Challenges

Previous researchers have noted frustrations at not having enough time to complete co‐analysis meaningfully, stating that there are no shortcuts to this process.^9^, ^11^, ^40^, ^48^, ^49^ This can be particularly challenging for doctoral research studies which are often time‐bound. In this project, each stage of the analysis process took longer than anticipated. The familiarization and coding phase of analysis was completed over 11 meetings across 10 weeks. The searching for and reviewing themes phases took place over 4 meetings across 4 weeks. The report writing phase took 8 hours over 5 weeks. Thus, the entire process took almost 5 months to complete from start to finish. Future projects wishing to involve people with lived‐experience in qualitative data analysis are therefore advised to schedule sufficient time to allow for this process to be meaningfully co‐produced.

Further restrictions were imposed on the ways of working with the contributor by ethical considerations. For instance, in line with ethical approvals, data had to be viewed by all researchers on the University Campus in accordance with principles of data security. This therefore limited the location of meetings to the University Campus, where other locations could have provided easier access, a greater sense of comfort and confidence and may have gone some way to ameliorating the power imbalance between academic researchers and contributors. Alleviating this geographic restriction on data could also have implications for resources; had the contributor been able to familiarize themself with transcripts in their own home, this would have eliminated travel expenses for this stage. This would, of course, need to be balanced with the need to preserve security of the data, and so future researchers should consider whether honorary contracts may enable this flexibility in terms of remote access to data.

An initial concern that LH held prior to embarking on this co‐analysis was the need to follow an inductive approach to data analysis, ensuring to stay true to the data. Others have noted the difficulty with this, reporting that the similarities in experiences between contributors and participants can often blur the boundaries.^40^, ^50^ Despite this initial concern, the incorporation of reflexive notes from both academic and lay researchers helped to mitigate this. This ensured that both the contributor and LH adopted a critical stance of their own experiences and perspectives, discussing these where relevant, but separating them from the experiences of participants. Future researchers involving people with lived‐experience in analysis are encouraged to advocate reflexivity throughout. This study and previous researchers^40^ found value in providing a structured approach to this.

### 3.2 Benefits

It is well established that triangulation of perspectives in data analysis can lead to a richer, more detailed analysis of qualitative data.^51^, ^52^, ^53^ Previous researchers have noted the particular benefits that a lived‐experience perspective can bring, for instance leading to new and exciting lines of inquiry and providing alternative perspectives to data.^9^, ^11^, ^40^, ^50^, ^54^ Sweeney et al (2013) noted this is particularly useful to ensure that alternate and competing explanations have been considered before consensus is reached. As previously noted, the contributions of the contributor at the coding phase did not often differ greatly from the codes ascribed by academic researchers. However, there were some instances in which codes differed and these were primarily in relation to the role of the system in helping prisoners. This is interesting, as previous lay researchers have noted that academic researchers often automatically take into account the restraints of the working environment, whereas lay researchers may often be unaware or choose to ignore these, and therefore look at findings with a more open mind (see Cowley et al, 2019). Moreover, whilst the content of codes ascribed by LH and the contributor often captured the same themes, the labelling of codes often differed greatly. This raises an important reflection as the reporting of later themes often centred around the language used by the contributor, which tended to be more closely aligned with the language typically used by participants. This contribution is therefore likely to improve the readability of subsequent reports for lay audiences, and in some cases may even more closely reflect the experiences of participants than the labels ascribed by academic researchers.

Related to the differing interpretations of data, involving contributors in the analysis may have gone some way to removing the ‘lens’ with which the academic researchers viewed the truth. Beresford (2005) states that ‘the shorter the distance there is between direct experience and its interpretation (as for example can be offered by user involvement in research and particularly user controlled research), then the less distorted, inaccurate and damaging resulting knowledge is likely to be’. (p. 7). Other researchers have noted how involving people with lived‐experience has ameliorated the impact of academic researchers own biases and worldview beyond the capabilities of reflexive practice.^11^, ^40^

Involving contributors in the analysis process can also be seen to improve the rigour of the process in other ways. For instance, previous researchers have noted that contributors can facilitate a constant comparison process, whereby a back and forth process of clarification is achieved.^11^, ^40^ This was achieved in the current study by returning to transcripts and drawings to help clarify candidate themes and subthemes. Further, it is generally agreed that multiple coding increases external validity of qualitative analysis.^9^, ^55^, ^56^ This suggests therefore that the analysis of these data may hold greater external validity than if data were coded only by LH, as is often the case in doctoral research.

### 3.3 Contributor reflections

I have enjoyed the process and the chance to work collaboratively and not just be consulted when the work has been done. For instance, I have enjoyed working on coding data together and comparing and contrasting different viewpoints and interpretations.

On a personal level, it has been beneficial for my own well‐being to feel listened to, respected and I value contributing to an important study using my own lived‐experience. I felt valued throughout, and I enjoyed the process. It felt good to use the experiences I gained in prison, such as being a prison ‘listener’ for nearly three years, where I was trained to provide emotional support to other prisoners. I was reminded of the difficulties both other prisoners and myself have had in expressing feelings in a hostile environment and the need to be safe around this.

I think other researchers would benefit from involving ex‐prisoners in their research because it brings a different perspective. I think researchers need to give consideration to training needs and the emotional impact of being involved in research for people who have experienced stigma and discrimination due to having been in prison. I felt it was a worthwhile experience and would encourage other researchers to involve contributors using similar methods.

## 4 CONCLUSIONS AND RECOMMENDATIONS

**FIGURE 2:**
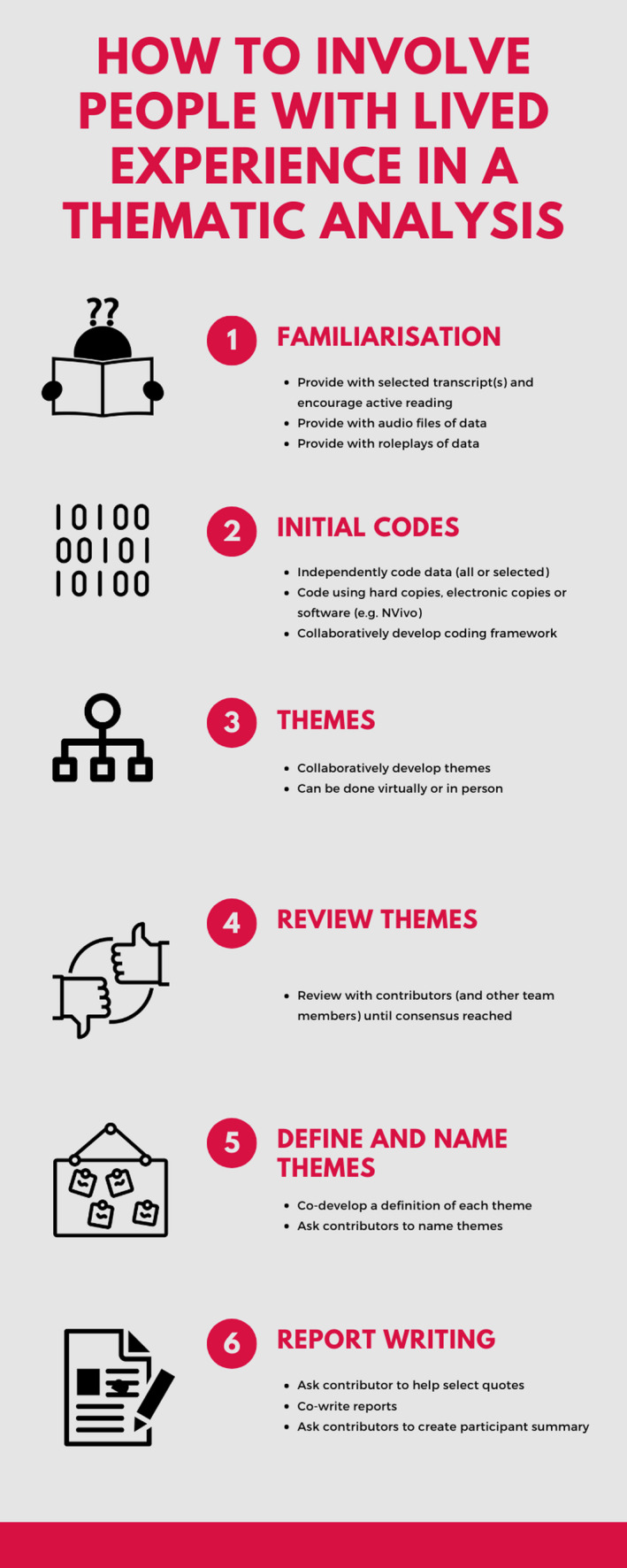
How to involve people with lived‐experience in a thematic analysis

The level of patient and public involvement in this study can be seen as akin to that of collaboration and co‐production, as defined by Hughes and Duffy (2018),^57^ with members of the public being involved as members of the research team and contributing to key decisions regarding research processes and findings. Specifically, contribution from a person with lived‐experience of the phenomena being studied was sought at each of the six stages of a Braun and Clarke (2006) thematic analysis. Figure 2 outlines some ways in which contributors can be involved at each stage of the analysis, drawn both from the experiences of this study, along with previous literature.

**FIGURE 3:**
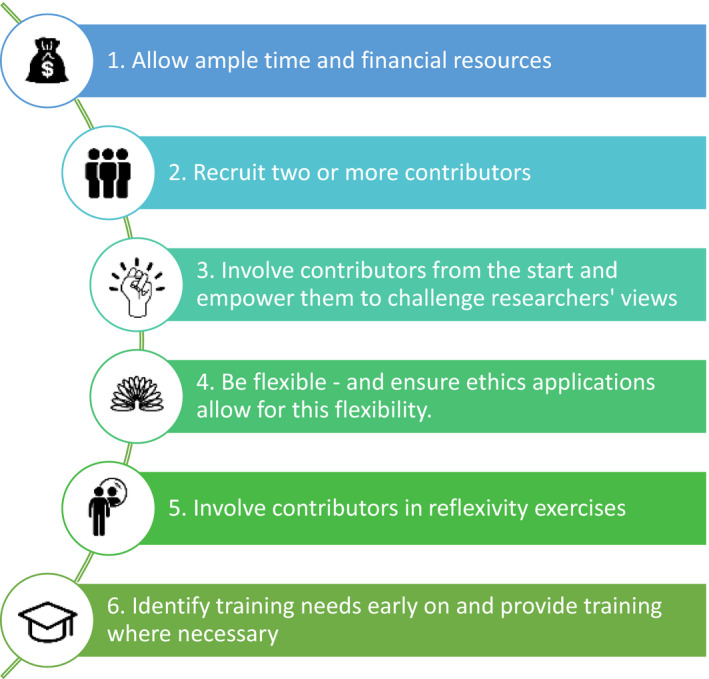
Recommendations

In addition to this, a number of recommendations are made to facilitate the meaningful involvement of people with lived‐experience in a thematic analysis and to avoid tokenistic involvement (Figure 3). First, researchers are encouraged to allow ample time and resources to meaningfully involve contributors in qualitative analysis. This will allow for meaningful involvement of contributors at several phases of the analysis process and will allow for multiple coding of the entire data set. Second, consideration should be given to recruiting two or more people with lived‐experience to collaborate on the analysis process. This may help to redress issues of power imbalance that could exist between academic and lay researchers. Where this is not possible, it is important to ensure that sole contributors feel empowered to challenge the views and perceptions of academic researchers. Establishing rapport with contributors and involving them from the start of research projects can help to achieve this. Third, involving contributors with analysis requires a great deal of flexibility. For instance, depending on the needs of contributors, additional consideration may need to be given to things such as providing access to data in alternative formats such as printed copies instead of electronic copies, access requirements for meetings and issues with literacy. It is important to make sure that ethical approvals allow for this flexibility in working. Fourthly, it is essential to ensure contributors are involved in all reflexivity processes. This ensures that analysis remains true to the data, and pre‐existing viewpoints and conceptions are not imposed on the data set. It can help to provide structured guidance with this. Fifthly, researchers are advised to assess training needs early on and provide training where necessary to give contributors a basic understanding of qualitative research and analysis.

This paper adds to the current literature by providing a detailed report, which adheres to GRIPP2 reporting guidelines^55^ of involving somebody with lived‐experience in a qualitative analysis. This paper has shown that it was both possible and beneficial to meaningfully engage in a substantial patient and public involvement co‐analysis as part of doctoral study, drawing on limited financial resources. Such an endeavour has proven to benefit the research in several ways, whilst also ensuring the research conducted adheres to social responsibilities outlined by the funders of this PhD. This project has highlighted the importance of academic researchers becoming more involved in co‐produced projects and gives recommendations and guidance for those wishing to do so.

## 5 PATIENT OR PUBLIC CONTRIBUTION

An individual with lived‐experience of being detained in prison was involved in designing this study, co‐analysing the data and preparing the manuscript for submission.

## CONFLICT OF INTEREST

The authors declare that the research was conducted in the absence of any commercial or financial relationships that could be construed as a potential conflict of interest.

## AUTHORS’ CONTRIBUTIONS

LH conducted all interviews. LH, PB, GH and JS were all involved in analysis. LH wrote the first draft of the manuscript. PB, GH, JS and DP wrote sections of the manuscript. All authors contributed to manuscript revision, read and approved the submitted version.

## Supporting information

Appendix S1

Appendix S2

Video S1

## Data Availability

The data that support the findings of this study are available on request from the corresponding author. The data are not publicly available due to privacy or ethical restrictions.
