# Supplementary material for: Involving an individual with lived‐experience in a co‐analysis of qualitative data
Source: Health Expect. 2021 Mar 31;24(3):766–75. doi: 10.1111/hex.13188 (PMC8235892; doi:10.1111/hex.13188)
Supplement: Supplementary file 1 — Appendix S1 [file HEX-24-766-s003.docx]

1. Reflexivity questions

Read over the transcript and make some notes on your initial thoughts of the transcript. Below are some questions to help you do this. You do not have to answer all of these questions – you don’t have to answer any of them if you don’t want, they’re just there as a guide. Just make some notes of what comes into your head when you’re reading the transcript for the first time. Do not attempt to code the transcript until you have done this. Do not worry about your notes being ‘messy’. They do not need to be fully developed ideas, simply thoughts that enter your head whilst reading the transcript. If you would prefer, you do not have to share these notes with me, so please be as honest and as raw as you can when making these notes. If you feel you need to read over the transcript a second time before coding then please feel free to do so.

1. How did the transcript make you feel?

2. What jumps out at you?

3. What surprised me?

4. What intrigued me?

5. What disturbed me?

6. What is this participant trying to accomplish? Why do they think their story is worth communicating?

7. Anything that resonates with your own experiences?

8. Anything that is very different from your own experiences?

9. Anything that you feel particularly critical of?

10. Anything you feel supportive of?

11. Any theories for how this transcript answers the research questions?

12. How ‘common-sense’ is their story?

13. How would you feel if you were in that situation? Is this different from or similar to how the participant feels and why might that be?

14. How does the participant make sense of the topic discussed? (e.g. talk about, characterise and understand what is going on)?

15. Why might they be making sense of their experiences in this way (and not another way)?

16. In what different ways do they make sense of the topic discussed?

17. What does the transcript tell you about the interview as an interactive process? E.g. are there moments when the interviewer / participant experiences strong emotions or sensations (e.g. relief, confusion, irritation, sweating, tightening of the stomach, uneasiness, anger etc.)? What might be going on there?

18. What assumptions is the participant making?

19. What do you see going on here?

20. What did you learn from this interview?
