# Supplementary material for: Involving an individual with lived‐experience in a co‐analysis of qualitative data
Source: Health Expect. 2021 Mar 31;24(3):766–75. doi: 10.1111/hex.13188 (PMC8235892; doi:10.1111/hex.13188)
Supplement: Supplementary file 2 — Appendix S2 [file HEX-24-766-s001.docx]

# GRIPP2 short form^55^

| Section and topic | Item | Reported on page no. |
| --- | --- | --- |
| 1: Aim | Report the aim of PPI in the study | 3 |
| 2: Methods | Provide a clear description of the methods used for PPI in the study | 5-10 |
| 3: Study results | Outcomes – report the results of PPI in the study, including both positive and negative outcomes | 10-12 |
| 4: Discussion and conclusions | Outcomes – comment on the extent to which PPI influenced the study overall. Describe positive and negative effects. | 10-12 |
| 5: Reflections / critical perspective | Comment critically on the study, reflecting on the things that went well and those that did not, so others can learn from this experience. | 10-12 |
